# Genomic Breeding Programs Realize Larger Benefits by Cooperation in the Presence of Genotype × Environment Interaction Than Conventional Breeding Programs

**DOI:** 10.3389/fgene.2020.00251

**Published:** 2020-04-21

**Authors:** Lu Cao, Huiming Liu, Han A. Mulder, Mark Henryon, Jørn Rind Thomasen, Morten Kargo, Anders Christian Sørensen

**Affiliations:** ^1^Center for Quantitative Genetics and Genomics, Aarhus University, Tjele, Denmark; ^2^Wageningen University & Research, Animal Breeding and Genomics, Wageningen, Netherlands; ^3^Danish Pig Research Centre, SEGES, Copenhagen, Denmark; ^4^School of Agriculture and Environment, The University of Western Australia, Crawley, WA, Australia; ^5^VikingGenetics, Randers, Denmark; ^6^SEGES, Aarhus, Denmark

**Keywords:** joint genetic evaluation, across-environment selection of sires, stochastic simulation, genetic gain, rate of inbreeding, long-term cooperation

## Abstract

Genotype × environment interaction (G × E) is of increasing importance for dairy cattle breeders due to international multiple-environment selection of animals as well as the differentiation of production environments within countries. This theoretical simulation study tested the hypothesis that genomic selection (GS) breeding programs realize larger genetic benefits by cooperation in the presence of G × E than conventional pedigree-based selection (PS) breeding programs. We simulated two breeding programs each with their own cattle population and environment. Two populations had either equal or unequal population sizes. Selection of sires was done either across environments (cooperative) or within their own environment (independent). Four scenarios, (GS/PS) × (cooperative/independent), were performed. The genetic correlation (*r*_*g*_) between the single breeding goal trait expressed in two environments was varied between 0.5 and 0.9. We compared scenarios for genetic gain, rate of inbreeding, proportion of selected external sires, and the split-point *r*_*g*_ that is the lowest value of *r*_*g*_ for long-term cooperation. Between two equal-sized populations, cooperative GS breeding programs achieved a maximum increase of 19.3% in genetic gain and a maximum reduction of 24.4% in rate of inbreeding compared to independent GS breeding programs. The increase in genetic gain and the reduction in rate of inbreeding realized by GS breeding programs with cooperation were respectively at maximum 9.7% and 24.7% higher than those realized by PS breeding programs with cooperation. Secondly, cooperative GS breeding programs allowed a slightly lower split-point *r*_*g*_ than cooperative PS breeding programs (0.85∼0.875 vs ≥ 0.9). Between two unequal-sized populations, cooperative GS breeding programs realized higher increase in genetic gain and showed greater probability for long-term cooperation than cooperative PS breeding programs. Secondly, cooperation using GS were more beneficial to the small population while also beneficial but much less to the large population. In summary, by cooperation in the presence of G × E, GS breeding programs realize larger improvements in terms of the genetic gain and rate of inbreeding, and have greater possibility of long-term cooperation than conventional PS breeding programs. Therefore, we recommend cooperative GS breeding programs in situations with mild to moderate G × E, depending on the sizes of two populations.

## Introduction

Genotype × environment interaction (G × E) is present in situations where a specific environmental variation has different effects on different genotypes (Falconer and Mackay, 1996b). Environmental variation can be physical, chemical, biological, behavior patterns, or life events (Ottman, 1996). G × E is often illustrated by the unparallel norms of reaction (Lynch and Walsh, 1998). It can lead to scaling effect including different variances, and reranking effect of genotypes between environments. Reranking is often measured by the genetic correlation (*r*_*g*_) between a trait expressed in two different environments (Falconer, 1952). An *r*_*g*_ less than unity indicates the presence of G × E. For instance, G × E exists for milk production in Holstein dairy cattle with *r*_*g*_ ranging from 0.6 to 1 between environments within and between countries (Konig et al., 2005; Nauta et al., 2006; Haile-Mariam et al., 2008; Huquet et al., 2012; Hugo et al., 2017). The *r*_*g*_ of functional traits, such as fertility, tends to be lower than that of production traits (*r*_*g*_ = 0.33 ∼ 0.74) (Haile-Mariam et al., 2008; Hugo et al., 2017). When G × E exists, it makes good sense to utilize the best combinations of genotypes and environments for more efficient animal production because G × E implies that genotypes differ in genetic potential of adapting to variable environments (Falconer and Mackay, 1996c; Montaldo, 2001). Therefore, from the perspective of breeding programs to achieve more efficient animal production, it is essential to investigate the mechanisms that contribute to improving genetic gain and simultaneously decreasing inbreeding when G × E exists between different environments.

In many cases, different breeding programs are operated in different environments because of G × E. Moreover, cooperation of breeding programs, e.g., by using jointly each other’s sires, can result in larger genetic gain and lower rate of inbreeding. The cooperation discussed here refers to the combination of joint-evaluation and across-environment selection. Cooperation of breeding programs in the presence of G × E depends on the *r*_*g*_ between environments. In the era before genomic selection (GS), Banos and Smith (1991) proposed that selection of Holstein sires across countries would achieve the maximum genetic response in each country when *r*_*g*_ was above 0.7 or 0.8. Smith and Banos (1991) added that small populations benefited from combined selection, whereas large populations also benefited but less when the initial genetic means were the same. Mulder and Bijma (2006) found that cooperation between two equal-sized breeding programs using conventional evaluation and selection strategies was possible in the first generations with *r*_*g*_ as low as 0.4 to 0.6, but only possible in the long term with *r*_*g*_ as high as 0.8 to 0.9, which was called the split-point *r*_*g*_. The split-point *r*_*g*_ is the lowest value of *r*_*g*_ for long-term cooperation. In other words, if *r*_*g*_ is lower than split-point *r*_*g*_ then each breeding program selects animals from the external population only in the short-term, while if *r*_*g*_ is higher than split-point *r*_*g*_ then each breeding program selects animals from the external population in the short and also long term. Thus, when the split-point *r*_*g*_ is lower, it indicates greater probability of long-term cooperation. It is evident that the level of G × E influences the decision of running cooperative or independent breeding programs. The above-mentioned studies using pedigree-based selection (PS) found improvement in genetic gain by cooperation but none of them investigated the impact of cooperation on the rate of inbreeding. While our study attempted to fill this gap of rate of inbreeding and investigated the mechanisms of benefiting from cooperation using GS in the presence of G × E.

Genomic selection increases genetic gain and reduces rate of inbreeding compared to the conventional methods (Schaeffer, 2006; Daetwyler et al., 2007; Buch et al., 2012). We hypothesized that this also applies to GS implemented in situations where across-environment genetic evaluation and selection of external breeding animals takes place. Genotypes for single nucleotide polymorphisms (SNP) are more likely to be well replicated across environments than individuals or their close relatives (Hayes et al., 2016). This enables GS to increase the accuracies of domestic animals and external animals and reduce the difference in accuracy between them compared to PS. Consequently, GS exploits larger variation in the distributions of estimated breeding values (EBV) of both domestic candidates and external candidates and brings two distributions closer to each other compared to conventional PS evaluation, thus increases genetic gain and pulls the split-point *r*_*g*_ to a lower numeric value, i.e., has a greater probability of long-term cooperation. Inbreeding is expected to be reduced by the increased reference population that contains more genetic diversity and by the selection of external animals across environments with slightly different selection. In addition, at the split-point *r*_*g*_ it is expected to observe the minimum rate of inbreeding. Because at the split-point *r*_*g*_ it is an open breeding system with the greatest number of ancestors who have made long-term genetic contributions to the current generation among all levels of *r*_*g*_. The greatest number of ancestors results in a minimum rate of inbreeding, according to the relationship between long-term genetic contributions from a generation of ancestors and rate of inbreeding of the current population: $\Delta \text{F}=1/4\left(1-\alpha \right)\sum r_{i}^{2}$, where ΔF is the rate of inbreeding of the current generation, α is the constant departure from Hardy-Weinberg proportions, *r_i_* is the long-term genetic contribution of ancestor *i* (Wray and Thompson, 1990; Bijma and Woolliams, 2000; Woolliams et al., 2015). In view of these rationale, we hypothesized that GS breeding programs would realize larger improvements in genetic gain and rate of inbreeding by cooperation, and have greater probability for long-term cooperation in the presence of G × E than conventional PS breeding programs. To test this hypothesis, we stochastically simulated two breeding programs that resembled two dairy cattle breeding programs carried out in two different environments each with their own cattle population. Two breeding programs had either equal or unequal population sizes. Selection was for a single trait, which could also be regarded as a multi-trait selection index. We also varied the *r*_*g*_ of the selected trait expressed in two environments and the heritability (*h*^2^) of that trait.

## Materials and Methods

### Design

We used stochastic simulation to estimate improvements in genetic gain and inbreeding and the probability of long-term cooperation made by two cooperative GS or PS breeding programs in the presence of G × E. For this purpose, we designed four scenarios each with two interacting breeding programs that resembled two dairy cattle breeding programs carried out in two different environments.

#### Across-Environment Genomic Evaluation and Across-Environment Selection (AG-AS)

Both breeding programs adopted across-environment single-step genomic best linear unbiased prediction (ssGBLUP) for all animals and across-environment selection for sires in generations *t* = 6…20. This scenario simulated two cooperative GS breeding programs. “Cooperation” in this study was defined as the situation that one population eventually selected sire(s) from the other population in generations *t* = 6…20 based on across-environment genetic evaluation.

#### Within-Environment Genomic Evaluation and Within-Environment Selection (WG-WS)

Both breeding programs adopted within-environment ssGBLUP for all animals and within-environment selection for sires in generations *t* = 6…20. This scenario simulated two independent GS breeding programs. It provided the reference to estimate the benefits of cooperation made by **AG-AS** compared to the independent GS breeding programs.

#### Across-Environment Pedigree-Based Evaluation and Across-Environment Selection (AP-AS)

Both breeding programs adopted across-environment pedigree-based BLUP (PBLUP) for all animals and across-environment selection for sires in generations *t* = 6…20. This scenario simulated two cooperative conventional breeding programs. It was compared to **AG-AS** for the capability of benefiting from cooperation in the presence of G × E either with PBLUP or ssGBLUP.

#### Within-Environment Pedigree-Based Evaluation and Within-Environment Selection (WP-WS)

Both breeding programs adopted within-environment PBLUP for all animals and within-environment selection for sires in generations *t* = 6…20. This scenario simulated two independent conventional breeding programs. It provided the reference to estimate the benefits of cooperation made by **AP-AS** compared to independent PS breeding programs.

For all four scenarios, two breeding programs — having their own environment (E1/E2) and cattle population — had either equal (1000:1000) population sizes or unequal (400:1600) population sizes. The population size was determined by the number of selected reproductive females. The selected trait was controlled by 2,000 biallelic quantitative trait loci (QTL). Each breeding program aimed to improve the breeding goal trait in its own environment (Trait 1/Trait 2) and selected sires and dams by truncation selection on the EBV of its own environment. Trait 1 and Trait 2 in the base population had equal initial genetic means of 0, additive genetic variances ($\sigma_{a}^{2}$) of 1.0 and *h*^2^ ranging from 0.1 to 0.5. The *r*_*g*_ between Trait 1 and Trait 2 varied from 0.5 to 0.9 representing different levels of G × E between E1 and E2. Phenotypes were only observed for females, which resembled milk production. Each scenario was replicated 50 times and each replicate was simulated for 20 discrete generations (*t* = 0, 1…20). Each generation took one unit of time that consisted of a series of related events, namely culling, update of reproductive cycles, selection, mating and sampling offspring.

### Simulation Procedure

#### Generation −3000 to −1: Founder Population

A common founder population was simulated using QMSim software (Sargolzaei and Schenkel, 2009) for all replicates of all scenarios, to establish linkage disequilibrium (LD) information between markers and QTL. It was based on the LD profile of Danish Red Dairy Cattle population in order to have a genetic architecture that resembles a real population. This reference genome-wide LD profile refers to 6,581 animals with 43,621 markers (unpublished). The founder population was simulated for 3,000 discrete generations, and the numbers of both sexes always remained the same, and it was always random mating with replacement between males and females. The detailed simulation set-up for founder population applied in this study was same as the “diverse” breeding program tailed for Red dairy cattle in Thomasen et al. (2020), which intensively elaborates information such as the number and the length of chromosomes, the initial numbers and distribution of QTL and SNP markers, set-up regarding expansion and bottleneck of the population size, the rule of descendants inheriting alleles, the selection of the final QTL and SNP markers, and so on. At generation *t* = **−**1, 2,000 QTL and 40,000 SNP loci were obtained; chromosomes from 300 male and 300 female founders were gathered by sex to form 30 paternal and 30 maternal pools each with 600 chromosomes. Chromosomes were subsequently sampled from these pools to establish genotypes of base animals. To sum up, the effective population size of the founder population is 600 (Oldenbroek, 2007).

#### Generation 0: Base Population

Simulation specifically for each scenario was initiated at generation *t* = 0 by sampling genotypes from those above-mentioned chromosome pools to form the base population consisting of 200 males and 4,000 females. For each base animal, two chromosomes for each pair of chromosomes were randomly sampled without replacement from the corresponding chromosome pool of its sex. Then the pair of sampled chromosomes were replaced before sampling for the next base animal. Base animals were designed to be non-inbred and unrelated to each other by assigning each of them unique alleles at 6,000 IBD (identical by descent) marker loci, which included 200 IBD markers equidistantly distributed within each chromosome and never involved in selection. Each base animal’s contribution to their descendant generations was traced and true inbreeding of descendants relative to the base population was inferred based on these 6,000 IBD markers.

Base animals were randomly appointed to E1 and E2, and the numbers of appointed males and females to either environment were determined by the population size. For the *i*^*th*^ base animal in each environment, the phenotype of the breeding goal trait, *p_i_*, was calculated as *p*_*i*_ = *a*_*i*_ + *e*_*i*_, where *a_i_* was that base animal’s true breeding value (TBV) and *e_i_* was the residual environmental value. In particular, TBV for the breeding goal trait (*a_i_*) as well as TBV for the “unexpressed” trait (*a*_*iu*_) that was only expressed in the other environment was simulated simultaneously, both of which were calculated as the sum of 2,000 QTL effects. The effects of each QTL on the two traits followed a bivariate normal distribution, $\left[\begin{array}{c} a_{i}\\ a_{iu} \end{array}\right]∼N\left(\left[\begin{array}{c} 0\\ 0 \end{array}\right],\;\left[\begin{array}{cc} 1 & r_{g}\\ r_{g} & 1 \end{array}\right]\right)$, and all effects were subsequently scaled to achieve an initial total genetic variance-covariance matrix of $\left[\begin{array}{cc} 1 & r_{g}\\ r_{g} & 1 \end{array}\right]$ (i.e., for each trait $\sigma_{qtl}^{2}=\sigma_{a}^{2}=1$, the additive QTL variance explained all genetic variance). *e_i_* was obtained by sampling from e∼*N*(0, 1/*h*^2^−1). As a result, the phenotypes of the breeding goal trait of base animals in each environment had a mean of 0 and a standard deviation of 1/*h*.

#### Generation 1: Random Selection From Base Population

Parents in generation *t* = 1 were randomly selected from base animals within environments with the selected proportion of 50%. In generations *t* = 1…5, only selected sires were genotyped.

In generations *t* = 1…20, selected males were evenly and randomly mated with selected females. QTL transmitted from parents to offspring as per Mendelian rules without newly generated mutation after the founder population was established. QTL effects on both traits were the same as for base animals, but allele frequency at each QTL could change due to selection and drift. TBV for each trait for each animal born in generations *t* = 1…20 was the sum of two allelic effects over all QTL. The residual environmental values and phenotypes were obtained as for base animals. To maintain population sizes, each selected female produced three offspring with a probability of 0.5 for both male and female offspring.

#### Generation 2 to 5: Separate Breeding Using Within-Environment Evaluation and Selection

In generations *t* = 2…5, each breeding program was carried out independently in their own environment using within-environment truncation selection for both sexes based on within-environment PBLUP.

#### Generation 6 to 20: “Scenarios”

In generations *t = 6…20*, four scenarios were carried out with different genetic evaluation strategies or different selection strategies of sires as above-mentioned. But dams were always selected within-environments irrespective of scenarios. All animals were genotyped prior to selection. Table 1 lists the simulation information of two breeding programs in scenario **AG-AS** in full details. Except for the genetic evaluation strategies and the selection strategies of sires in generations *t* = 6…20, other all scenarios shared the same details as **AG-AS**.

**TABLE 1:** Details of breeding programs in two environments for scenario **AG-AS**^1^.

	EPS^2^	UPS^2^
	(♀*E*1^3^:♀*E*2^3^ = 1000:1000)	(♀*E*1:♀*E*2 = 400:1600)
		
No. of discrete generations	20	
Selection unit (♂, 1∼5 generation)	within-environment	
Selection unit (♂, 6∼20 generation)	across-environment	
Selection unit (♀)	within-environment	
Selection criterion (2∼5 generation)	within-environment PBLUP-EBV	
Selection criterion (6∼20 generation)	across-environment ssGBLUP-EBV	
Genotyped (1∼5 generation)	selected sires	
Genotyped (6∼20 generation)	all animals	
No. of selected sires per generation (1∼5 generation)	50 (E1); 50 (E2)	20^4^ (E1); 80^4^ (E2)
No. of selected sires per generation (6∼20 generation)	50^4^ (E1); 50^4^ (E2)	
No. of selected dams per generation	1000 (E1); 1000 (E2)	400 (E1); 1600 (E2)
No. of offspring per dam	3	
Heritability of the breeding goal trait	0.1, 0.2, 0.3, 0.4, 0.5	0.3^5^
Genetic correlation (r_*g*_)	0.8, 0.825, 0.85, 0.875, 0.9^6^	0.5, 0.6, 0.7, 0.8, 0.9

*^1^**AG-AS**, the scenario of across-environment genomic evaluation and across-environment selection for sires. ^2^EPS/UPS, the situation of equal/unequal population sizes. ^3^E1/E2, environment 1/environment 2. ^4^Numbers of selected sires in UPS situation in generations t = 1…5 different from in generations t = 6…20 was to ensure a consistent selected proportion between two breeding programs in each generation. ^5^Only heritability of 0.3 was considered for UPS, as we assumed no interaction between heritability and EPS/UPS. ^6^EPS situation considered only a small range of r_g_ because our preliminary results showed neither of two environments selected external sire(s) when r_g_ was lower than 0.8.*

### Genetic Evaluation

Genetic evaluation was executed by DMU version 6 (Madsen and Jensen, 2014) in generation *t* = 2…20. The models used in all scenarios can be summarized as follows:

$$\left[\begin{array}{c} {\text{y}}_{1}\\ {\text{y}}_{2} \end{array}\right]=\left[\begin{array}{cc} {\text{X}}_{1} & 0\\ 0 & {\text{X}}_{2} \end{array}\right]\left[\begin{array}{c} \beta_{1}\\ \beta_{2} \end{array}\right]+\left[\begin{array}{cc} {\text{Z}}_{1} & 0\\ 0 & {\text{Z}}_{2} \end{array}\right]\left[\begin{array}{c} {\text{a}}_{1}\\ {\text{a}}_{2} \end{array}\right]+\left[\begin{array}{c} {\text{e}}_{1}\\ {\text{e}}_{2} \end{array}\right]$$

where **y_i_** was the vector of observations of *i*th trait, *i* = 1, 2; **β_i_** was the vector of generation as the fixed effect in the *i*th environment, the number of levels of which increased by one every generation; **a_i_** was the vector of additive genetic effects of *i*th trait; and **e_i_** was the vector of residual effects of *i*th trait. **X_i_** and **Z_i_** were the incidence matrices connecting **β_i_** and **a_i_** to **y_i_**, respectively. It was assumed that $\left[\begin{array}{c} {\text{a}}_{1}\\ {\text{a}}_{2} \end{array}\right]∼N\left(0,\left[\begin{array}{cc} \sigma_{a_{1}}^{2} & r_{g}\sigma_{a_{1}}\sigma_{a_{2}}\\ r_{g}\sigma_{a_{1}}\sigma_{a_{2}} & \sigma_{a_{2}}^{2} \end{array}\right]⊗\text{H}\right)$ and $\left[\begin{array}{c} {\text{e}}_{1}\\ {\text{e}}_{2} \end{array}\right]∼N\left(0,\left[\begin{array}{cc} \sigma_{e_{1}}^{2} & 0\\ 0 & \sigma_{e_{2}}^{2} \end{array}\right]⊗\text{I}\right)$, where **H** was the matrix of additive genetic relationships among individuals in the pedigree if it was PBLUP, or the unified genetic relationship matrix of all genotyped and non-genotyped animals in the pedigree if it was ssGBLUP (Legarra et al., 2014); **I** was the identity matrix; ⊗ was the symbol for the Kronecker product of two matrices; $\sigma_{a_{i}}^{2}$ was the additive genetic variance of *i*th trait; *r*_*g*_σ_*a*_1__σ_*a*_2__ was the additive genetic covariance between two traits; $\sigma_{e_{i}}^{2}$ was the residual variance of *i*th trait (Henderson and Quaas, 1976). For ssGBLUP, the **H**-matrix requires the genomic covariance matrix of breeding values, **G,** for genotyped animals, which was obtained by the second method proposed by VanRaden (2008). Residual covariance between two traits was zero, because each animal stayed in one environment and therefore only expressed the breeding goal trait in that environment. When the evaluation was within environment, the *r*_*g*_ was set to 0, equivalent to running simultaneously two univariate models.

### Genetic Gain, Rate of Inbreeding, and Split-Point Correlation

The indicators used to evaluate each breeding program were genetic gain per generation, rate of inbreeding per generation and the split-point *r*_*g*_.

*Genetic gain* per generation was computed for each replicate as the regression coefficient of mean TBV of animals on generation (*t* = 10…20). The presented genetic gain was the average over 50 replicates. The unit of genetic gain was the genetic standard deviation of the breeding goal trait in the base population.

*Rate of inbreeding* per generation was calculated for each replicate as Δ*F* = 1 − *exp*(*b*_*ln*(1−*F*_*t*_), *t*_), where ΔF was rate of inbreeding, *F*_*t*_ was the mean inbreeding coefficient at generation *t*, *b*_*ln*(1−*F_t_*), *t*_ was the regression coefficient of the natural logarithm of (1 − *F*_*t*_) on generation (*t* = 10…20). Because the inbreeding coefficient at any generation *t* referred to the base population is

$$F_{t}=1-{\left(1-\Delta F\right)}^{t}\left(\mathrm{Falconer and Mackay, 1996a}\right)$$

Therefore,

$$ln\left(1-F_{t}\right)=ln\left(1-\Delta F\right)\times t$$

which deduces that *ln*(1 − Δ*F*) is the regression coefficient of *ln*(1 − *F*_*t*_) on generation *t*. *F*_*t*_ was derived based on the average inbreeding coefficient of those 6,000 fore-mentioned IBD loci. Inbreeding at each IBD locus was defined as the probability that two alleles at that locus for a randomly selected animal out of the population are IBD. The presented rate of inbreeding was the average over 50 replicates. In addition, the ancestors who had made long-term genetic contributions to the current generation were traced and counted to help interpret the occurrence or absence of the minimum rate of inbreeding.

We evaluated genetic gain and rate of inbreeding per generation instead of per unit of time. Because the biological risks of the negative influence of inbreeding, namely inbreeding depression and deleterious alleles, as well as its balancing processes, such as mutation, are more relevant in the context of generation being the time scale (Daetwyler et al., 2007). Although the rates per generation numerically equalized the rates per unit of time in this study. Moreover, results regarding genetic gain and rate of inbreeding were based on generation 10 to 20 to avoid the unsteadiness caused by build-up of pedigree information (Dekkers, 1992) and reference information for genomic prediction, and reduction of genetic variance due to selection (Bulmer, 1971). Hereinafter improvements in genetic gain and rate of inbreeding by cooperation will be expressed as percentage of increment of cooperative scenarios over their corresponding independent scenarios. In details, the improvement in genetic gain and rate of inbreeding by **AG-AS** will be respectively calculated as [(Δ*G*_*AG*−*AS*_ − Δ*G*_*WG*−*WS*_)/Δ*G*_*WG*−*WS*_] and [(Δ*F*_*AG*−*AS*_ − Δ*F*_*WG*−*WS*_)/Δ*F*_*WG*−*WS*_] in percentage terms. While by **AP-AS**, they will be respectively [(Δ*G*_*AP*−*AS*_ − Δ*G*_*WP*−*WS*_)/Δ*G*_*WP*−*WS*_] and [(Δ*F*_*AP*−*AS*_ − Δ*F*_*WP*−*WS*_)/Δ*F*_*WP*−*WS*_] in percentage terms.

*Split-point r*_*g*_ was determined for scenario **AG-AS** and **AP-AS** after computing the proportion of sires from the external environment in all sires selected by each environment in generations *t = 6…20*. The split-point *r*_*g*_ is the lowest value of *r*_*g*_ for long-term cooperation. In other words, above the split-point *r*_*g*_, one breeding program kept selecting sires from the external environment without decrease in the proportion of external sires till generation 20.

### Software

The simulations were run using ADAM (Pedersen et al., 2009), a software to stochastically simulate breeding programs for animals and plants.

## Results

### Improvements in Genetic Gain and Rate of Inbreeding

The following sections presented findings for the situation that two environments had equal population sizes. These results were only presented for E1; results from E2 were similar to E1.

**FIGURE 1:**
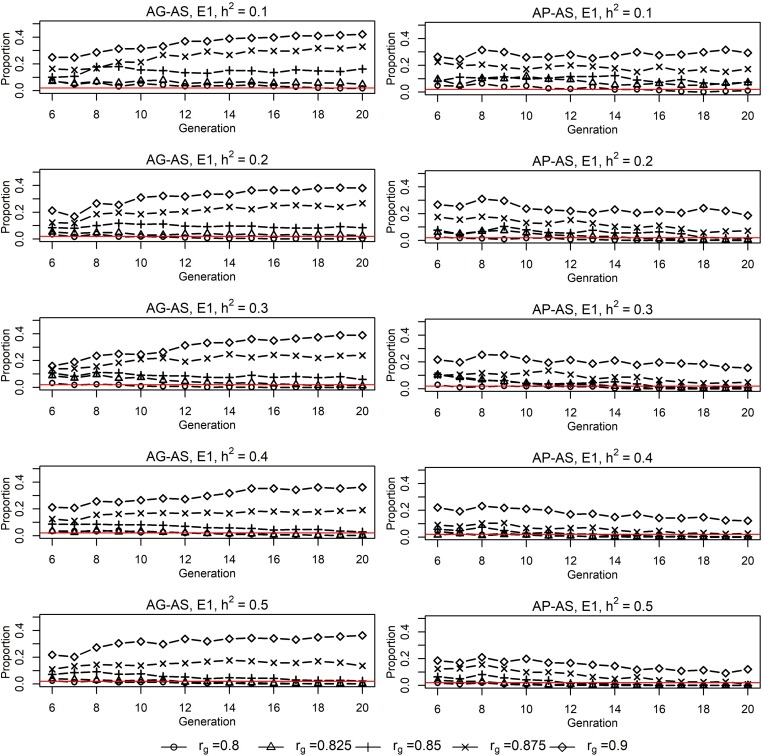
Proportions of sires from E2^1^ in sires selected by E1^1^ for EPS^2^ situation for **AG-AS**^3^ and **AP-AS**^3^. ^1^E1/E2, environment 1/environment 2. ^2^EPS, situation that two environments had equal population sizes (1000:1000). ^3^**AG-AS**, scenario of across-environment genomic evaluation and across-environment selection for sires; **AP-AS**, scenario of across-environment pedigree-based evaluation and across-environment selection for sires. The red horizontal line at *Y* = 0.02 acts as the baseline, below which it implies that E1 selected no sire from E2.

Genomic selection breeding programs realized larger increase in genetic gain by cooperation than conventional PS breeding programs (Table 2). Scenario **AG-AS** realized genetic gain ranging from 0.50 ± 0.04 to 0.79 ± 0.04, while **AP-AS** realized genetic gain between 0.31 ± 0.04 and 0.51 ± 0.04. It was almost always **AG-AS** > **WG-WS** > **AP-AS** > **WP-WS** in terms of the ranking of genetic gain. **AG-AS** increased up to 19.3% in genetic gain relative to **WG-WS**, while **AP-AS** increased up to 9.6% relative to **WP-WS**. **AG-AS** had significantly larger (*P* < 0.0001) increase in genetic gain than **AP-AS** given the same *h*^2^ and the same *r*_*g*_. At most **AG-AS** realized 9.7% higher increase than **AP-AS** at *h*^2^ = 0.1 and *r*_*g*_ = 0.9. Also note that, at *h*^2^ = 0.3 and *r*_*g*_ = 0.8, **AG-AS** obtained improvements in genetic gain with an increase of 1.2% and in rate of inbreeding with a reduction of 7.1% relative to **WG-WS**, without selecting any external sire in generations *t* = 10…20 (Figure 1). Following this, we ran an additional scenario, **AG-WS** (across-environment genomic evaluation and within-environment selection for sires) for all levels of *r*_*g*_ combined with *h*^2^ of 0.3. It turned out to be intermediate between **AG-AS** and **WG-WS** in terms of genetic gain and rate of inbreeding (not shown).

**TABLE 2:** Genetic gain per generation (ΔG^1^) and rate of inbreeding per generation (ΔF) realized in E1^2^ for EPS^3^ situation ^4^.

		ΔG (ICI^5^)						ΔF (ICI)					
h^2^	r_*g*_	AG-AS^6^		WG-WS^6^	AP-AS^6^		WP-WS^6^	AG-AS		WG-WS	AP-AS		WP-WS
0.1	0.8	0.50	(2.3%)	0.49	0.31	(−0.8%)	0.31	0.0100	(−14.5%)	0.0117	0.0130	(1.5%)	0.0128
	0.825	0.51	(5.4%)		0.33	(6.4%)		0.0096	(−17.9%)		0.0132	(3.1%)	
	0.85	0.53	(9.3%)		0.32	(2.3%)		0.0093	(−20.5%)		0.0129	(0.6%)	
	0.875	0.55	(11.8%)		0.34	(9.2%)		0.0097	(−17.1%)		0.0138	(7.6%)	
	0.9	0.58	(19.3%)		0.34	(9.6%)		0.0106	(−9.4%)		0.0124	(−3.4%)	
0.2	0.8	0.60	(3.7%)	0.58	0.38	(2.5%)	0.37	0.0097	(−4%)	0.0101	0.0115	(−6.8%)	0.0123
	0.825	0.59	(2.6%)		0.38	(1.2%)		0.0090	(−10.9%)		0.0119	(−3%)	
	0.85	0.62	(7%)		0.38	(1.2%)		0.0086	(−14.9%)		0.0116	(−5.9%)	
	0.875	0.64	(10.7%)		0.39	(3.7%)		0.0087	(−13.9%)		0.0125	(1.8%)	
	0.9	0.65	(13.5%)		0.40	(6.2%)		0.0088	(−12.9%)		0.0109	(−11.1%)	
0.3	0.8	0.64	(1.2%)	0.64	0.42	(3.9%)	0.41	0.0091	(−7.1%)	0.0098	0.0109	(−5.7%)	0.0115
	0.825	0.65	(2.3%)		0.43	(5.7%)		0.0082	(−16.3%)		0.0118	(2.1%)	
	0.85	0.66	(4.1%)		0.42	(2.6%)		0.0082	(−16.3%)		0.0102	(−11.6%)	
	0.875	0.69	(8.1%)		0.42	(3.6%)		0.0078	(−20.4%)		0.0114	(−1.3%)	
	0.9	0.70	(10.6%)		0.44	(7.8%)		0.0081	(−17.3%)		0.0097	(−15.4%)	
0.4	0.8	0.69	(3.5%)	0.67	0.46	(3.8%)	0.44	0.0085	(−8.6%)	0.0093	0.0101	(−2.6%)	0.0103
	0.825	0.69	(2.4%)		0.46	(3.8%)		0.0083	(−10.8%)		0.0105	(2%)	
	0.85	0.70	(4.6%)		0.46	(4.6%)		0.0077	(−17.2%)		0.0095	(−7.5%)	
	0.875	0.72	(7.3%)		0.45	(2%)		0.0074	(−20.4%)		0.0107	(3.8%)	
	0.9	0.75	(12.4%)		0.48	(7.9%)		0.0076	(−18.3%)		0.0095	(−7.7%)	
0.5	0.8	0.72	(2.3%)	0.71	0.48	(0.7%)	0.48	0.0086	(−4.4%)	0.0090	0.0093	(−11.1%)	0.0104
	0.825	0.73	(2.7%)		0.47	(−1.7%)		0.0081	(−10%)		0.0104	(−0.3%)	
	0.85	0.74	(4.3%)		0.48	(0.4%)		0.0074	(−17.8%)		0.0091	(−13.2%)	
	0.875	0.76	(7.9%)		0.49	(1%)		0.0068	(−24.4%)		0.0098	(−6.3%)	
	0.9	0.79	(12.1%)		0.51	(5.5%)		0.0078	(−13.3%)		0.0088	(−16.1%)	

*^1^ The unit of ΔG was the genetic standard deviation of the breeding goal trait in the base population. ^2^E1/E2, environment 1/environment 2. ^3^EPS, the situation that two environments had equal population sizes (1000:1000). ^4^The standard error was 0.03 ∼ 0.05 for ΔG and 0.0009 ∼ 0.0037 for ΔF. ^5^ICI, improvement in ΔG or ΔF of cooperative scenarios over the corresponding independent scenarios (percentages shown in parentheses), calculated as [(ΔG_AG−AS_−ΔG_WG−WS_)/ΔG_WG−WS_] or [(ΔF_AG−AS_−ΔF_WG−WS_)/ΔF_WG−WS_] in percentage terms for **AG-AS** scenarios, and [(ΔG_AP−AS_−ΔG_WP−WS_)/ΔG_WP−WS_] and [(ΔF_AP−AS_−ΔF_WP−WS_)/ΔF_WP−WS_] for **AP-AS** scenarios. For example at h^2^ = 0.1 and r_g_ = 0.9, **AG-AS** scenario realized 19.3% increase [(0.58–0.49)/0.49] in genetic gain compared to **WG-WS**; at h^2^ = 0.5 and r_g_ = 0.875, **AG-AS** scenario realized 24.4% reduction [(0.0068–0.0090)/0.0090] in rate of inbreeding compared to **WG-WS**. ^6^**AG-AS**, scenario of across-environment genomic evaluation and across-environment selection for sires; **WG-WS**, scenario of within-environment genomic evaluation and within-environment selection for sires; **AP-AS**, scenario of across-environment pedigree-based evaluation and across-environment selection for sires; **WP-WS**, scenario of within-environment pedigree-based evaluation and within-environment selection for sires.*

Genomic selection breeding programs realized larger reduction in rate of inbreeding by cooperation than conventional PS breeding programs (Table 2). Scenario **AG-AS** realized rate of inbreeding ranging from 0.0068 ± 0.0016 to 0.0106 ± 0.0025, while **AP-AS** realized rate of inbreeding between 0.0088 ± 0.0027 and 0.0138 ± 0.0036. In most cases, it was **AG-AS** < **WG-WS** < **AP-AS** < **WP-WS** in terms of the numeric ranking of rate of inbreeding. Throughout all combinations of *h*^2^ and *r*_*g*_, **AG-AS** decreased the rate of inbreeding up to 24.4% relative to **WG-WS**, while **AP-AS** decreased the rate of inbreeding up to 16.1% relative to **WP-WS**. By comparison, **AG-AS** had significantly larger (*P* < 0.00001) reduction in rate of inbreeding than **AP-AS** given the same *h*^2^ and the same *r*_*g*_. At most, **AG-AS** realized 24.7% larger reduction in rate of inbreeding than **AP-AS** at *h*^2^ = 0.1 and *r*_*g*_ = 0.875, where **AG-AS** favorably realized a reduction in inbreeding rate while **AP-AS** unfavorably realized an increase in inbreeding rate relative to their respective independent scenarios.

**FIGURE 2:**
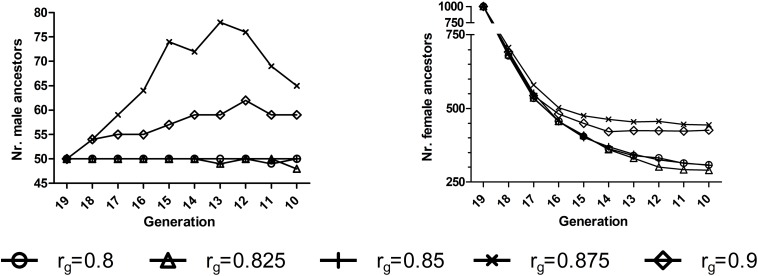
Numbers of ancestors who had made long-term genetic contributions to 3,000 offspring born at generation 20 in E1^1^ for a randomly selected replicate for EPS^2^ situation for **AG-AS**^3^ (*h*^2^ = 0.3). ^1^E1, environment 1.^ 2^EPS, the situation that two environments had equal population sizes (1000:1000). ^3^**AG-AS**, scenario of across-environment genomic evaluation and across-environment selection for sires.

Special attention was focused on **AG-AS** because it displayed the strongest capacity of increasing genetic gain and reducing rate of inbreeding. Within **AG-AS** genetic gain increased as *r*_*g*_ increased independent of the heritability, with a few exceptions (Table 2). For all preset levels of *h*^2^, the minimum rate of inbreeding appeared at *r*_*g*_ of 0.85∼0.875 that will be proven as the split-point *r*_*g*_ of **AG-AS** in the next paragraph. At this split-point *r*_*g*_ of 0.85∼0.875, we found that the greatest number of ancestors had made long-term genetic contributions to the current generation. For instance, the numbers of male and female ancestors reached a maximum at *r*_*g*_ = 0.875, the split-point *r*_*g*_ for *h*^2^ = 0.3, in generations *t* = 10…18 for E1 (Figure 2). In fact, the minimum rate of inbreeding of **AP-AS** appeared at *r*_*g*_ of 0.9 (Table 2), which will also be proven as the split-point *r*_*g*_ of **AP-AS** subsequently.

### Split-Point Correlation Between Short-Term and Long-Term Cooperation

**FIGURE 3:**
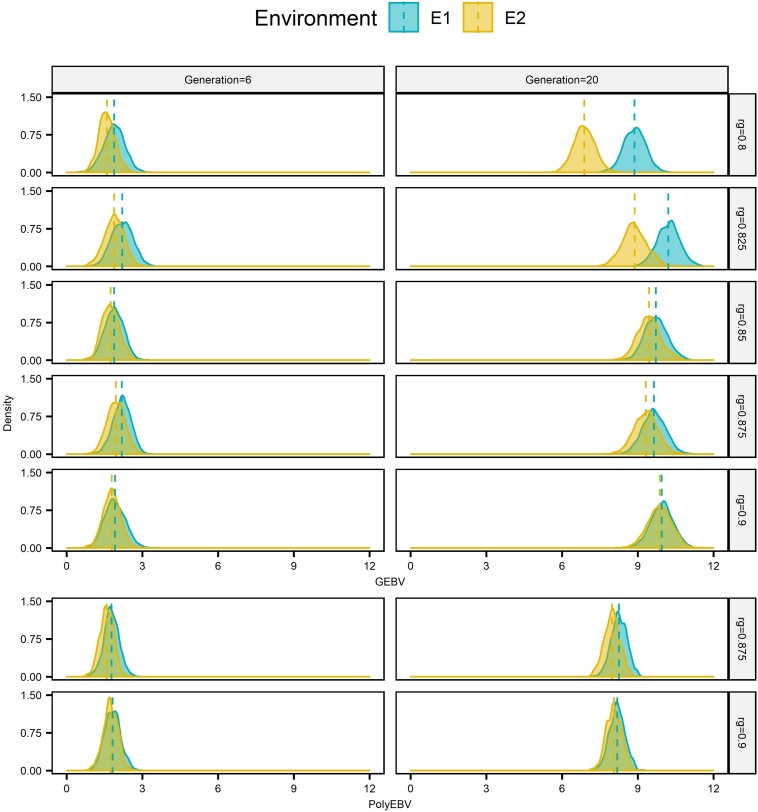
Distributions of EBV of male candidates from two sources^1^ at generation 6 and 20 for E1^2^ based on one single randomly selected replicate for EPS^3^ situation for scenario **AG-AS**^4^ and **AP-AS**^4^ (*h*^2^ = 0.3). ^1^Candidates from E1 and E2 are in blue and in yellow, respectively. ^2^E1/E2 refers to environment 1/environment 2. ^ 3^EPS refers to the situation that two environments had equal population sizes (1000:1000). ^4^**AG-AS**, scenario of across-environment genomic evaluation and across-environment selection for sires; **AP-AS**, scenario of across-environment pedigree-based evaluation and across-environment selection for sires. Vertical dashed lines mark distribution means.

Genomic selection breeding programs showed a slightly lower split-point *r*_*g*_ between short-term cooperation and long-term cooperation than conventional PS breeding programs (0.85∼0.875 vs. ≥0.9, Figure 1). For **AG-AS,** E1 begun cooperation by using sires from E2 for breeding when *r*_*g*_≥0.8. It selected 0 to 42.12% of sires from E2 over generations with *h*^2^ of the breeding goal trait varing from 0.1 to 0.5. The proportion of sires from E2 increased as *r*_*g*_ increased, regardless of *h*^2^. However, the trend of selecting sires from E2 in the long term, i.e., long-term cooperation, differed among different *r*_*g*_. When *r*_*g*_ was less than 0.85∼0.875 selection of sires from E2 diminished and even vanished quickly in the first 5 generations. While when *r*_*g*_ was larger than 0.85∼0.875 sustainable long-term cooperation became possible and sires from E2 were always selected by E1 to maximize genetic gain. Therefore, 0.85∼0.875 was identified as *r*_*g*_ that split up short-term cooperation and long-term cooperation between two cooperating GS breeding programs with equal population sizes. In contrast for **AP-AS**, the split-point *r*_*g*_ between two cooperating PS breeding programs with equal population sizes was ≥ 0.9 and therefore slightly higher than in **AG-AS**. The trend of proportion of selected external sires was determined by the genetic levels and variances of EBV in two populations over generations (Figure 3). In comparison, given the same *r*_*g*_, say *r*_*g*_ = 0.875, GS retained larger variances of EBV of both domestic and external candidates thus resulted in a larger portion of external candidates in the top ranking (the proportion increased from 14% in generation 6 to 22% in generation 20 using **AG-AS**, while decreased from 10% to 2% using **AP-AS**). It can be concluded that genomic selection decreases the split-point *r*_*g*_ from 0.9 to 0.85–0.875 compared to pedigreed-based selection, because of gaining larger variances in EBV of domestic and external sires.

### Unequal Population Sizes

Genomic selection breeding programs realized larger increase in genetic gain by cooperation than conventional PS breeding programs (Table 3). It was obviously **AG-AS** ≥ **WG-WS** > **AP-AS** or **WP-WS** in terms of the ranking of the genetic gain for both E1 and E2. For E1 that had the small population size**, AG-AS** increased up to 51.7% in genetic gain relative to **WG-WS,** and **AP-AS** increased up to 22% relative to **WP-WS**. For E2 with the large population size, **AG-AS** increased up to 4.3% relative to **WG-WS,** and **AP-AS** increased up to 0.5% relative to **WP-WS**. **AG-AS** had significantly larger increase in the genetic gain than **AP-AS** given the same *h*^2^ and the same *r*_*g*_ for both E1 (*P* = 0.0006) and E2 (*P* = 0.002), with the maximum difference between the increases realized by **AG-AS** and **AP-AS** being 29.7% for E1 and 4.8% for E2 both at *r*_*g*_ = 0.9. These results revealed that cooperation was more beneficial for the small population in terms of genetic gain.

**TABLE 3:** Genetic gain per generation (ΔG^1^) and rate of inbreeding per generation (ΔF) realized in E1^2^ and E2^2^ for UPS^3^ situation (*h*^2^ = 0.3) ^4^.

		ΔG (ICI^5^)						ΔF (ICI)					
	r_*g*_	AG-AS^6^		WG-WS^6^	AP-AS^6^		WP-WS^6^	AG-AS		WG-WS	AP-AS		WP-WS
E1 (small)	0.5	0.49	(3.9%)	0.47	0.34	(−1.9%)	0.34	0.0095	(4.7%)	0.0091	0.0091	(−7.3%)	0.0098
	0.6	0.49	(4.7%)		0.33	(−2.5%)		0.0090	(−0.9%)		0.0091	(−7.6%)	
	0.7	0.52	(10.4%)		0.35	(2.5%)		0.0073	(−19.5%)		0.0092	(−6.8%)	
	0.8	0.62	(33.3%)		0.38	(10.7%)		0.0070	(−22.6%)		0.0088	(−10.2%)	
	0.9	0.71	(51.7%)		0.42	(22%)		0.0094	(3.5%)		0.0129	(30.7%)	
E2 (large)	0.5	0.73	(0%)	0.73	0.45	(−3%)	0.46	0.0097	(0.3%)	0.0097	0.0120	(−9.2%)	0.0132
	0.6	0.73	(0.7%)		0.46	(−0.3%)		0.0095	(−2.2%)		0.0126	(−4.2%)	
	0.7	0.73	(0.8%)		0.46	(−0.1%)		0.0094	(−3.2%)		0.0122	(−7.9%)	
	0.8	0.74	(2.4%)		0.46	(0.5%)		0.0092	(−4.8%)		0.0128	(−2.7%)	
	0.9	0.76	(4.3%)		0.46	(−0.5%)		0.0093	(−3.6%)		0.0127	(−3.6%)	

*^1^ The unit of ΔG was the genetic standard deviation of the breeding goal trait in the base population. ^2^E1/E2, environment 1/environment 2. ^3^UPS, the situation that two environments had unequal population sizes (400:1600). ^4^The standard error of E1 was 0.03 ∼ 0.5 for ΔG and 0.0010 ∼ 0.0052 for ΔF; while the standard error of E2 was 0.03 ∼ 0.05 for ΔG and 0.0010 ∼ 0.0028 for ΔF. ^5^ICI, improvement in ΔG or ΔF of cooperative scenarios over the corresponding independent scenarios (percentages shown in parentheses), calculated as [(ΔG_AG−AS_−ΔG_WG−WS_)/ΔG_WG−WS_] or [(ΔF_AG−AS_−ΔF_WG−WS_)/ΔF_WG−WS_] in percentage terms for **AG-AS** scenarios, and [(ΔG_AP−AS_−ΔG_WP−WS_)/ΔG_WP−WS_] and [(ΔF_AP−AS_−ΔF_WP−WS_)/ΔF_WP−WS_] for **AP-AS** scenarios. For example at r_g_ = 0.9, **AG-AS** scenario realized 51.7% increase [(0.71 − 0.47)/0.47] in genetic gain compared to **WG-WS** in E1; **AP-AS** scenario realized 3.6% reduction [(0.0127 − 0.0132)/0.0132] in rate of inbreeding compared to **WP-WS** in E2. ^6^**AG-AS**, scenario of across-environment genomic evaluation and across-environment selection for sires; **WG-WS**, scenario of within-environment genomic evaluation and within-environment selection for sires; **AP-AS**, scenario of across-environment pedigree-based evaluation and across-environment selection for sires; **WP-WS**, scenario of within-environment pedigree-based evaluation and within-environment selection for sires.*

**FIGURE 4:**
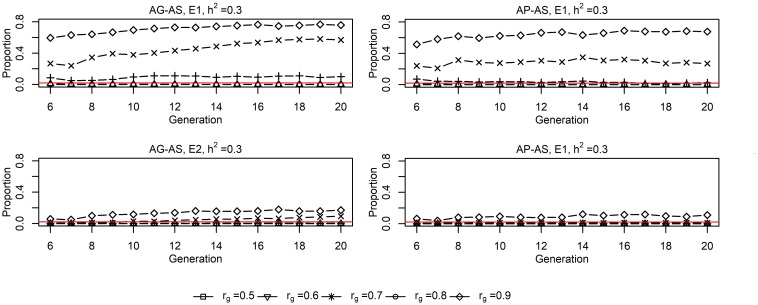
Proportions of sires from the other environment in sires selected by E1^1^ and E2^1^ for UPS^2^ situation for **AG-AS**^3^ and **AP-AS**^3^ (*h*^2^ = 0.3). ^1^ E1/E2, environment 1/environment 2. ^2^ UPS, situation that two environments had unequal population sizes (400:1600). ^3^ **AG-AS**, scenario of across-environment genomic evaluation and across-environment selection for sires; **AP-AS**, scenario of across-environment pedigree-based evaluation and across-environment selection for sires. The red horizontal line at *Y* = 0.02 acts as the baseline, below which it implies that one environment selected no sire from the other environment.

Genomic selection breeding programs allowed for a lower split-point *r*_*g*_ between short-term cooperation and long-term cooperation than PS breeding programs, with 0.6∼0.7 vs. 0.7∼0.8 for the small population and 0.7∼0.8 vs. 0.8∼0.9 for the large population (Figure 4). Using either **AG-AS** or **AP-AS**, E1 started to use sires from E2 at a lower *r*_*g*_ than E2 did from E1, and E1 selected a larger proportion of sires from E2 than E2 did from E1. Both together suggested the small population was more inclined for cooperation than the large population in order to maximize genetic gain. Moreover, GS breeding programs lowering the split-point *r*_*g*_ especially for small population also showed that the small population benefited from cooperation already at substantial degrees of GxE.

However, based on existing experimental design and results, we found no pattern that GS breeding programs realized larger reduction in rate of inbreeding by cooperation than conventional PS breeding programs (Table 3). It showed that **AG-AS** ≤ **WG-WS** < **AP-AS** < **WP-WS** in terms of the numeric ranking of rate of inbreeding was only apparent for E2. Even so, the reduction in inbreeding rate realized by **AG-AS** relative to **WG-WS** was not always greater than that realized by **AP-AS** relative to **WP-WS** given the same *h*^2^ and the same *r*_*g*_. Nevertheless, other patterns of interest were found. First, the inbreeding rates realized in E1 by implementing **AG-AS** and **AP-AS** both decreased first and then increased as *r*_*g*_ increased. At the level of *r*_*g*_ where the minimum inbreeding rate appeared, we also observed the largest numbers of male and female ancestors who made long-term contributions to the current generation (not shown). Secondly, E1 displayed greater variation of inbreeding rates across all levels of *r*_*g*_ than E2 by implementing either **AG-AS** or **AP-AS**. Then note the generally much higher rate of inbreeding in E2 than in E1 when using either **WP-WS** or **AP-AS**, while rather similar rates of inbreeding were observed in E2 and E1 when using either **WG-WS** or **AG-AS**. Also note the sharp increased rates of inbreeding at *r*_*g*_ = 0.9 in E1 by **AP-AS** (0.0129) and **AG-AS** (0.0094).

## Discussion

Our findings supported our hypothesis that GS breeding programs benefit more from cooperation regarding genetic gain and rate of inbreeding and have greater possibility of long-term cooperation in the presence of G × E than conventional PS breeding programs. Cooperation here referred to the combination of joint genetic evaluation and selection of sires across two environments. Otherwise no cooperation meant two populations remained independent by executing a completely separate breeding program within its own environment. GS increases accuracy therefore increasing genetic gain and decreasing rate of inbreeding. Cooperation with using external bulls from the other environment as candidates, increased the selection intensity thus increased genetic gain and also introduced more genetic diversity therefore decreased rate of inbreeding. Genomic selection increased the benefits of cooperation so that achieving larger increases in genetic gain, larger reductions in rate of inbreeding and a lower split-point genetic correlation. In short, genomic selection shows larger probability of long-term cooperation compared to conventional pedigree-based breeding programs in the presence of G × E. Hence, we recommend cooperative GS breeding programs with mild to moderate G × E depending on the sizes of two populations.

### Improvements in Genetic Gain and Rate of Inbreeding

Genomic selection breeding programs realize larger improvement in genetic gain by cooperation than PS breeding programs (Täubert et al., 2011; Thomasen et al., 2020) because of increased selection intensity and accuracy. The increased selection intensity due to incorporating external animals as candidates (Banos and Smith, 1991; Smith and Banos, 1991; Mulder and Bijma, 2006) also applies to PS breeding programs. The increased accuracy is, however, specific to GS breeding programs and mainly attributes to joint genetic evaluation of animals, which increases the reference population and therefore the accuracy. To check the contribution of joint genomic evaluation, we simulated **AG-WS** in which the cooperation included only joint genomic evaluation, but selection was within environment. Results showed that even such a “single-layer” cooperation strategy was also beneficial for genetic gain and rate of inbreeding, as reported by Slagboom et al. (2019). Subsequently when introducing the second level of cooperation, i.e. across-environment selection of sires, there emerges an additional aspect of increased accuracy: the variances of EBV of both domestic and external candidates increase more under GS breeding strategy than those under PS breeding strategy (Figure 3). This enables GS breeeding strategy not only to result in a higher probability for external sires to be top-ranked and selected, but also highly likely to increase selection intensity in turn due to the larger selection differential. Therefore, in the presence of mild G × E (*r*_*g*_ ≥ 0.8) between two equally sized populations from two different environments, cooperative GS breeding programs are superior with a greater ability of increasing genetic gain than cooperative conventional PS breeding programs, due to increased accuracy of both domestic and external sires and increased selection intensity.

Genomic selection breeding programs realizing larger improvement in rate of inbreeding by cooperation is directly supported by the finding that **AG-AS** had significantly larger reduction in rate of inbreeding than **AP-AS**. This supportive proof is backed up by two underlying findings. First, GS breeding programs realized lower rate of inbreeding than conventional breeding programs either with cooperative breeding strategy or independent breeding strategy (Thomasen et al., 2020), because GS increases estimation accuracy of the Mendelian sampling term, thus allows for better differentiation within families and leads to lower coselection of sibs. More precisely, GS captures partly the Mendelian sampling term for non-phenotyped individuals and allows more balanced selection (utilizing between- and within-family variation simultaneously) than PS, which does not capture the Mendelian sampling term at all for non-phenotyped individuals (Daetwyler et al., 2007). Secondly, cooperative breeding programs realized lower rate of inbreeding than independent breeding programs using either GS or PS due to the import of genetic material from the external environment that introduces more genetic diversity. Because of the larger proportion of external sires selected, GS keeps its advantage in rate of inbreeding with cooperation and achieves a larger reduction in rate of inbreeding than cooperative PS breeding programs. So in the presence of mild G × E (*r*_*g*_ ≥ 0.8) between two equally sized populations, cooperative GS breeding programs have greater capacity of slowing down rate of inbreeding than conventional cooperative PS breeding programs, because of more families selected and more genetic diversity introduced.

**AG-AS** did not only always have the highest genetic gain and lowest rate of inbreeding than other strategies, irrespective of *r*_*g*_ and *h*^2^. Meanwhile **AG-AS** showed two obvious patterns within itself. First, the genetic gain increased as *r*_*g*_ increased due to more genetically related traits providing more valuable information and resulting in higher estimation accuracy. Second, the rate of inbreeding first increased then decreased as *r*_*g*_ increased with the minimum rate of inbreeding appearing at *r*_*g*_ of 0.85∼0.875 — the split-point *r*_*g*_ for two equally sized populations, which was well explained by the number of ancestors of long-term contribution. The rate of inbreeding did not trend up all the way like the genetic gain, suggesting that the weaker G × E is not necessarily the better if the balance between inbreeding and genetic gain is desired.

### Improvement in Possibility of Long-Term Cooperation

Genomic selection breeding programs have greater probability of long-term cooperation compared to conventional breeding programs, which was supported by the lower split-point *r*_*g*_ of **AG-AS** than that of **AP-AS** (0.85∼0.875 vs ≥0.9). Given a level of *r*_*g*_, a trade-off exists between the increased difference of genetic levels of two populations and the increased variation of EBVs (increased accuracy) of candidates from these two populations over time, as shown by Figure 3. As long as there is a net-benefit, cooperation will last, otherwise selection of external sires will reduce over time to zero. The result of this trade-off depends on *r*_*g*_ and determines how long the cooperation would last (Figure 1). Initiated with the same genetic levels for the breeding goal trait and the “unexpressed” trait in each environment, the genetic level of the breeding goal trait gradually surpassed the “unexpressed” trait with a superiority proportional to 1-*r*_*g*_, according to the different efficiency of direct selection and indirect selection (Falconer, 1952; Mulder and Bijma, 2006). The alleged “unexpressed” trait in one environment was just the breeding goal trait of the other environment. Thus, a higher *r*_*g*_ made two environments less distinct regarding the genetic level of the goal trait desired by one environment, which alleviated the difficulty for external sires to squeeze into the top ranking when across-environment selection was allowed. Meanwhile, higher *r*_*g*_ added more to the estimation accuracy that was already increased generation by generation due to increasingly accumulated pedigree-phenotypes-genotypes information over generations. This resulted in greater variations in the distributions of EBV of candidates from two sources, which indicated more likelihood for the overlap of two distributions. Therefore, a higher *r*_*g*_ performed better in the balancing act between the increased difference of genetic levels and the increased likelihood for the overlap between distributions of EBV of candidates from two sources. In comparison, GS is capable of retaining larger variations of EBV of domestic and external candidates than PS given the same *r*_*g*_, thus displays greater possibility of long-term cooperation. In previous studies, the similar case for short-term cooperation as the present study was also observed by Banos and Smith (1991), which reported that if *r*_*g*_ was less than 0.8 it would take at most 4 generations of selection until each population relied exclusively on its own bulls. Mulder and Bijma (2006) concluded that long-term cooperation between two equally sized environments was possible when *r*_*g*_ was higher than 0.80 to 0.90 for conventional BLUP evaluation using progeny testing. Smith and Banos (1991) reported the same finding as Mulder and Bijma (2006). The split-point *r*_*g*_ is the lowest value of *r*_*g*_ for long-term cooperation. The lower split-point *r*_*g*_ observed in GS breeding programs implies that, given G × E between two equally sized populations, genomic selection is more likely to keep a long-term sustainable cooperative breeding relationship than conventional selection strategies.

### Impacts of Unequal Population Sizes

The benefits of cooperation with GS compared to with PS were larger in the situation of unequal population sizes. The small population obtained much larger benefits in genetic gain by cooperation than the large population. These benefits for the small population resulted from a much larger selection intensity because of a much larger pool of selection candidates with cooperation than with independent breeding programs. Furthermore in the context of GS, the selection accuracy increased as well by cooperation because of a larger reference population and interchange of sires that created sufficient links between both populations. These benefits were smaller for the large population than for the small population, because the increase in selection intensity and accuracy were smaller (Schöpke and Swalve, 2016). As the small population improved, it gradually contributed genetic resources to the overall pool and attracted the large population to select animals from them (Banos and Smith, 1991). Because of the much larger benefits for the small population than for the large population, the small population started to use external sires at a lower *r*_*g*_ and therefore had a lower split-point *r*_*g*_ compared with the large population. Thus, when G × E is present between two unequally sized populations, GS breeding programs have greater ability to benefit from cooperation in terms genetic gain and more likelihood to keep long-term cooperation than conventional PS breeding programs, especially for the small population.

The inbreeding rates realized in the small environment E1 by **AG-AS** and **AP-AS** both decreased first and then increased as *r*_*g*_ increased. This pattern could be well explained using the number of ancestors with long-term contributions, in the same way as depicted for the situation with equal population sizes. The large environment E2 displayed smaller variation in inbreeding rates across all levels of *r_g_* than E1 by implementing either **AG-AS** or **AP-AS** due to selecting very few external sires (Figure 4), therefore being less affected by the genetic resource from the other environment. Daetwyler et al. (2007) reported that increased selection intensity substantially increases rate of inbreeding with BLUP but had no impact on the rate of inbreeding with genomic selection. This explained why we observed much higher rate of inbreeding in E2 than in E1 when using **WP-WS** or **AP-AS**, while quite similar rates of inbreeding in E2 and in E1 when using **WG-WS** or **AG-AS**. Due to our experimental design, two environments selecting same numbers of sires in generation *t* = 6…20, E2 had a higher selection intensity and a larger number of matings per sire than E1. E2 having a higher selection intensity than E1 was somewhat obscure for cooperative scenarios because of across-environment selection. But it was true since E2 had larger selection differential than E1 given the same level of *r_g_*, therefore effectively resulted in a higher selection intensity. With high *r*_*g*_, the rate of inbreeding of the large environment E2 determined that of the small environment E1, because E1 substantially relied on the importation of external sires from E2. This explained the sharply increased rate of inbreeding at *r*_*g*_ = 0.9 in E1 by either **AP-AS** or **AG-AS**. Nevertheless, if the effect of current experimental design was removed, we believe that GS breeding programs have greater ability to benefit from cooperation than conventional PS breeding programs in terms of the inbreeding rate given G × E present between two unequally sized populations.

### Generalization to Multiple Environments

We studied the situation with only two G × E environments. However, we think the above mechanisms revealed in this study can be generalized to the situation including three or more environments in the presence of G × E within each of the pairs of environments. No matter how many external populations there are or how complicated the G × E structure is among all pairs of environments, the population in operation of selection can always treat the other environments as a whole. Then the seemingly more sophisticated multi-environmental question becomes the dual-environmental question that we investigated in this study. Based on results in this study, the operating population can focus primarily on those environments that have moderate to high genetic correlations with the operating environment and treat those environments as one external environment. From an operational point of view, multiple-across country evaluation (MACE) of bulls (Schaeffer, 1994) enables worldwide selection of bulls and each breeding program can rank all available bulls according to the total merit index for the country of interest. VanRaden and Sullivan (2010) extended MACE to genomic information and Vandenplas et al. (2018) pointed out that summary statistics per SNP are required for joint evaluations, without the need of exchange of genomic and phenotypic data across countries. The broad concepts observed in this study are clearly applicable to multiple environments; from an operational point of view selection across environments can be done when using for instance MACE. There is no point in judging very detailed for every situation whether the genetic correlation is above or below the split-point genetic correlation, because the rankings of bulls will automatically indicate whether external bulls will be selected or not.

## Data Availability Statement

The datasets analyzed in this manuscript are not publicly available. Requests to access the datasets should be directed to cao.lu@mbg.au.dk.

## Author Contributions

AS and LC conceived this research, designed the scenarios and populations. LC, HL, JT, MK, and AS participated in the preliminary discussion of designed scenarios. LC performed the simulation and analyzed data with great help from HL. All authors involved in the interpretation of results. LC prepared the first draft. All authors contributed to revising the manuscript, especially HM and MH.

## Conflict of Interest

JT was employed by company VikingGenetics. MK was employed by company SEGES, Denmark. The remaining authors declare that the research was conducted in the absence of any commercial or financial relationships that could be construed as a potential conflict of interest.
